# Relationship between Obesity, Adipocytokines and Inflammatory Markers in Type 2 Diabetes: Relevance for Cardiovascular Risk Prevention

**DOI:** 10.3390/ijerph110404049

**Published:** 2014-04-14

**Authors:** Natasa Rajkovic, Miroslava Zamaklar, Katarina Lalic, Aleksandra Jotic, Ljiljana Lukic, Tanja Milicic, Sandra Singh, Ljubica Stosic, Nebojsa M. Lalic

**Affiliations:** Clinic for Endocrinology, Diabetes and Metabolic Diseases, Clinical Center of Serbia, Faculty of Medicine, University of Belgrade, Dr. Subotica 13, Belgrade 11000, Serbia; E-Mails: miraz@eunet.rs (M.Z.); katarina.s.lalic@gmail.com (K.L.); aleksandra.z.jotic@gmail.com (A.J.); ljlukic@eunet.rs (L.L.); icataca@gmail.com (T.M.); singh@eunet.rs (S.S.); bubichica@hotmail.com (L.S.); lalic.nm@gmail.com (N.M.L.)

**Keywords:** cardiovascular prevention, obesity, diabetes, adipocytokines, inflammatory markers

## Abstract

This study aimed to analyse the impact of obesity in type 2 diabetes (T2D) on adipocytokines (adiponectin, leptin and resistin) and inflammatory markers (TNF-α, IL-6 and hsCRP) as cardiovascular risk factors. A cross-sectional study comparing the basal levels of adipocytokines and inflammatory markers was done in 18 obese (BMI ≥ 30 kg/m^2^) (group A), 21 overweight (25 kg/m^2 ^≤ BMI < 30 kg/m^2^) (group B), 25 non-obese T2D patients (group C) and 15 non-obese controls (group D). The lowest levels of adiponectin and the highest levels of leptin, resistin, TNF-α, IL-6 and hsCRP were found in group A. Adiponectin levels were significantly lower, and resistin, TNF-α, and hsCRP levels were elevated in group C *vs.* D. However, leptin and IL-6 levels differed significantly between groups A and B, but not between groups C and D. Moreover, we found a significant negative correlation between adiponectin and TNF-α, but not with other markers, which was independent of the presence of obesity. In contrast, leptin and resistin correlated with the inflammatory markers, and this correlation was obesity-dependent. Our results suggest that obesity influences cardiovascular risk primarily through changes in leptin and resistin and less efficiently at the level of adiponectin.

## 1. Introduction

Previous studies have shown that obesity is associated with increased cardiovascular risk, especially when coexisting with type 2 diabetes (T2D) [1,2]. However, the mechanisms underlying this increased risk, either in obese T2D patients or in non-diabetic obese individuals, have not yet been clarified.

Moreover, it has been shown that in most studies aimed at reducing obesity by changes in lifestyle (diet and physical activity), lifestyle intervention had beneficial effects not only for preventing T2D but also when prevention of cardiovascular diseases (CVD) was concerned [3,4,5]. The mechanisms of this beneficial influence and their clinical relevance are poorly understood.

Some of the previous studies have suggested that adipocytokines and inflammatory markers might mediate the facilitating effect of obesity on the appearance of its comorbidities such as insulin resistance (IR), T2D and CVD [6].

Adiponectin levels were previously observed to be significantly diminished both in obesity and T2D [7,8,9]. Many studies described a significant negative correlation between adiponectin and the parameters of obesity [10,11]. In patients with T2D, adiponectin levels were lower than in age and BMI-matched non-diabetic men and women [12]. On the other hand, many studies showed that low adiponectin concentrations correlated with high plasma insulin and high IR, while adiponectin administration was found to suppress proinflammatory agents, e.g., TNF-α and IL-6, and to directly ameliorate endothelial dysfunction by increasing nitric oxide (NO) production [11,12,13,14].

Obesity is associated with hyperleptinaemia and leptin resistance. Leptin levels are elevated in proportion to the degree of adiposity, while the association of increased leptin with T2D is still unclear [15,16,17]. In cross-sectional studies, leptin levels have been associated with IR and the proinflammatory state that accompanies obesity [18]. At the same time, elevated leptin levels have been reported in patients with coronary artery disease (CAD) [19,20].

Resistin levels have been associated with obesity [20,21,22]. Moreover, some studies reported that resistin levels were higher in T2D subjects, but this increase could not be confirmed by other studies, or the relationship with IR or fasting insulin levels [23,24,25]. Also, clinical studies have revealed that plasma resistin levels correlate with markers of inflammation and might be predictive of coronary atherosclerosis [26].

Systemic inflammation could be the causative link between obesity, diabetes and cardiovascular diseases [27], as it induces inflammatory processes in the vessel wall. The chronic inflammatory response is characterized increases in the production of markers of inflammation *i.e.*, C-reactive protein (CRP), interleukin 6 (IL-6) and tumor necrosis factor alpha (TNF-α) [8,28]. High-sensitive CRP (hsCRP) has been proposed as an independent risk factor for CVD. Elevated concentrations of hsCRP were seen when obesity and diabetes were studied separately [27,29]. Several authors reported increased plasma IL-6 levels in obese patients as well as in patients with T2D [30,31,32,33]. However, some authors found a correlation between IL-6 and obesity and IR, but the results are still conflicting [34,35]. In obesity, chronically elevated TNF-α levels were detected and were also found to be associated with IR, increased plasma glucose and insulin levels [3,7] IRAS study confirmed that TNF-α is associated with T2D independently of adiposity [36]. Also, TNF-α was shown to correlate with carotid intima-media thickness (IMT) [37] and to be increased in premature CAD [38].

Therefore, in this study, we analysed the impact of obesity on changes in the levels of adipocytokines and inflammatory markers which might facilitate cardiovascular disease, in obese T2D patients. The analysis has also revealed the potential relevance of modulating these changes to cardiovascular risk prevention during lifestyle interventions targeting obesity.

## 2. Experimental Section

### 2.1. Subjects

In this study, we examined 65 T2D patients (mean age 57.8 years) and 15 control subjects. Diagnosis of T2D was confirmed using the oral glucose tolerance test (OGTT) in accordance with the WHO criteria [39]. The whole cohort of T2D patients was subdivided according to their body mass index (BMI) into three groups: obese patients (group A, *n* = 21, BMI ≥ 30 kg/m^2^), overweight patients (group B, *n* = 18, 25 kg/m^2^ ≤ BMI < 30 kg/m^2^) and lean patients (group C, *n* = 25, BMI < 25 kg/m^2^). Control subjects were healthy individuals with BMI < 25 kg/m^2^ (group D, *n* = 15). In addition to diet and exercise, the participants in the study were treated with metformin and sulfonylureas for hyperglycemia, either alone or in combination, as well as lipid lowering and antihypertensive therapy, all following standard recommendations [40]. Patients with clinically manifest CVD (except essential hypertension), renal diseases, urinary tract infections, thyroid disorders and liver function abnormalities, chronic inflammatory disorders, malignancies and psychiatric diseases were not included. The anthropometric and metabolic characteristics of patients and controls are shown in Table 1.

**Table 1:** Metabolic and anthropometric characteristics of the study subjects.

Characteristic	Group A *Obese T2D*	Group B *Overweight T2D*	Group C *Lean T2D*	Group D *Control Subjects*
*Number of subjectsMale/Female*	21 10/11	18 10/8	25 13/12	15 7/8
*Duration of T2D (y)* **^1^**	5.84 ± 0.1	7.3 ± 0.2	6.5 ± 0.1	/
*HbAlc (%)* **^1^**	6.3 ± 0.7	6.6 ± 0.4	6.5 ± 0.5	4.6 ± 0.4
*BMI (kg/ m^2^)* **^2^**	33.4 ± 2.8	28.3 ± 1.0	23.8 ± 1.5	22.7 ± 2.9
*Waist (cm)* **^3^** *Fat mass (kg)* **^4^**	110.4 ± 9.6 36.09 ± 7.0	100.7 ± 6.0 24.5 ± 6.0	85.5 ± 6.2 17.2 ± 4.5	82.4 ± 7.8 15.7 ± 6.8

Notes: ^**1**^ A *vs.* B *vs.* C: *p* = NS; **^2^** A *vs.* B *vs.* C *vs.* D: *p* < 0.05; A *vs.* B: *p* < 0.05; B *vs.* C: *p* < 0.01; C *vs.* D: *p* = NS; **^3^** A *vs.* B: *vs.* C *vs.* D: *p* < 0.01; A *vs.* B: *p* < 0.01; B *vs.* C: *p* < 0.01; C *vs.* D: *p* = NS; **^4^** A *vs.* B *vs.* C *vs.* D: *p* < 0.01; A *vs.* B: *p* < 0.01; B *vs.* C: *p* < 0.05; C *vs.* D: *p* = NS.

### 2.2. Measurement of Anthropometric and Metabolic Characteristics

Body mass index (BMI) was calculated as weight divided by height squared (kg/m^2^). Body composition and the amount and distribution of body fat (Fat Mass (FM)) were measured by bioelectrical impedance analysis techniques with bi-pedal electrode arrangement (Tanita Body Composition Analyzer, model TBF-300, manufacturer, Arlington Heights, IL, USA). The anthropometric and metabolic characteristics of patients and controls are shown in Table 1.

All the participants were studied at the Clinic for Endocrinology, Diabetes and Metabolic Diseases, Clinical Center of Serbia, Faculty of Medicine, University of Belgrade. The study was approved by the Ethics Committee of the Faculty of Medicine, University of Belgrade. All the participants provided written informed consent.

### 2.3. Laboratory Measurements

In T2D patients, antihyperglycemic, lipid lowering and antihypertensive medications were stopped 24 h before blood sampling. All the samples were taken after a 12 h fasting period.

Levels of total adiponectin (Mercodia, city, Sweden) as well as resistin, TNF-α, IL-6 (ALPCO Diagnostics, Windham, NH, USA), and hsCRP (ACL-Instrument Laboratory, Lexington, MA, USA) were measured in duplicate by an ELISA method using commercially available kits. Leptin (Linco, Seaford, DE, USA) and insulin levels (INEP, Beograd, Serbia) were determined with RIA. Glucose levels were measured by a glucose-oxidase method (Beckman Instruments, Fullerton, CA, USA). Homeostasis model assessment for insulin resistance evaluation (HOMA-IR) was calculated using the equation: fasting plasma insulin × glucose/22.5 [41].

### 2.4. Statistical Analysis

SPSS for Windows Version 16.0 (Chicago, IL, USA) was utilized for statistical evaluation. Values are expressed as mean ± SE. The Kolmogorov-Smirnov test was employed to compare normally distributed variables. One-way ANOVA with the Bonferroni post hoc multiple comparison test was used to compare differences between continuous variables in the groups, and Student T test was used to compare differences in variables between two groups. To compare differences in non-normally distributed variables the Kruskal-Wallis H test was used and the Mann-Whitney test was employed to compare differences between two groups. Spearman correlation coefficients were calculated to describe crude associations between variables (bivariate correlation) and the effect of potential confounding factors was tested in multivariate linear regression models. A *p*-value of <0.05 was considered statistically significant.

## 3. Results and Discussion

Metabolic and anthropometric characteristics of the subjects were summarized in Table 1. Patients were aged 40–70 years and matched for gender and duration of diabetes. In T2D the mean duration of diabetes was 6.6 years. All the diabetic patients had optimal glycemic control and HbA1c did not differ between the groups. BMI and fat mass significantly differed between the groups.

### 3.1. Evaluation of Insulin and HOMA-IR Levels

**Figure 1:**
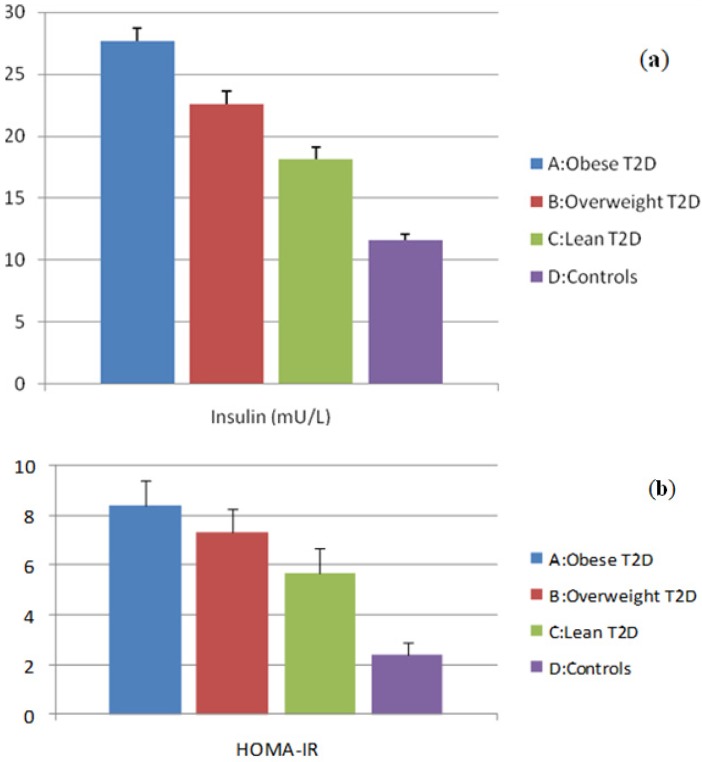
(**a**) Plasma levels of insulin; (**b**) Plasma levels of HOMA-IR.

Notes: A *vs*. B: *p* < 0.01; A, B *vs*. C: *p* < 0.05; C *vs*. D: *p* < 0.01.

Basal plasma insulin concentrations and HOMA-IR levels are shown in Figure 1a,b. The highest levels of insulin and HOMA-IR were found in group A and they were significantly higher than in group B. Both group A and group B had higher insulin and HOMA-IR levels compared with group C. There was a significant difference between group C and group D (Figure 1a,b).

### 3.2. Evaluation of Adipocytokine Levels

**Figure 2:**
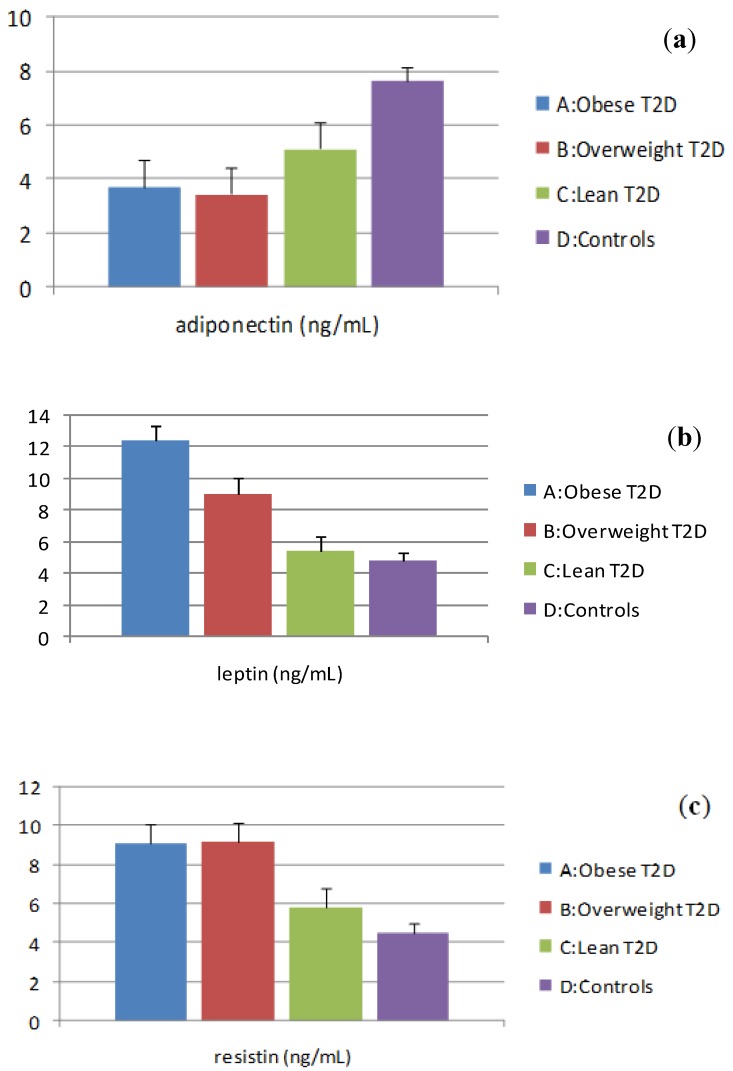
(**a**) Plasma levels of adiponectin; (**b**) Plasma levels of leptin; (**c**) Plasma levels of resistin.

Notes: In (**a**): A *vs*. B: *p* = NS; A, B *vs*. C: *p* < 0.01; C *vs*. D: *p* < 0.01; In (**b**): A *vs*. B: *p* < 0.01; A, B *vs*. C: *p* < 0.01; C *vs*. D: *p* = NS; In (**c**): A *vs*. B: *p* = NS; A, B *vs*. C: *p* < 0.01; C *vs*. D: *p* < 0.05.

Basal plasma concentrations of adipocytokines (adiponectin, leptin, resistin) are shown in Figure 2a–c. The lowest levels of adiponectin were found in group A while there was no difference between group A and group B. Both groups A and B had lower levels of adiponectin compared with group C. Adiponectin levels were significantly lower in group C when compared with group D (Figure 2a).

The highest levels of leptin were found in group A and they were significantly higher than in group B. Both groups A and B had higher leptin levels compared with group C and there was no difference between group C and group D (Figure 2b).

Moreover, the highest levels of resistin were found in group A but there was no difference between group A and group B. Both groups A and B had higher resistin levels compared with group C and the latter resistin levels significantly differed in comparison to group D (Figure 2c).

### 3.3. Evaluation of Inflammatory Marker Levels

**Figure 3:**
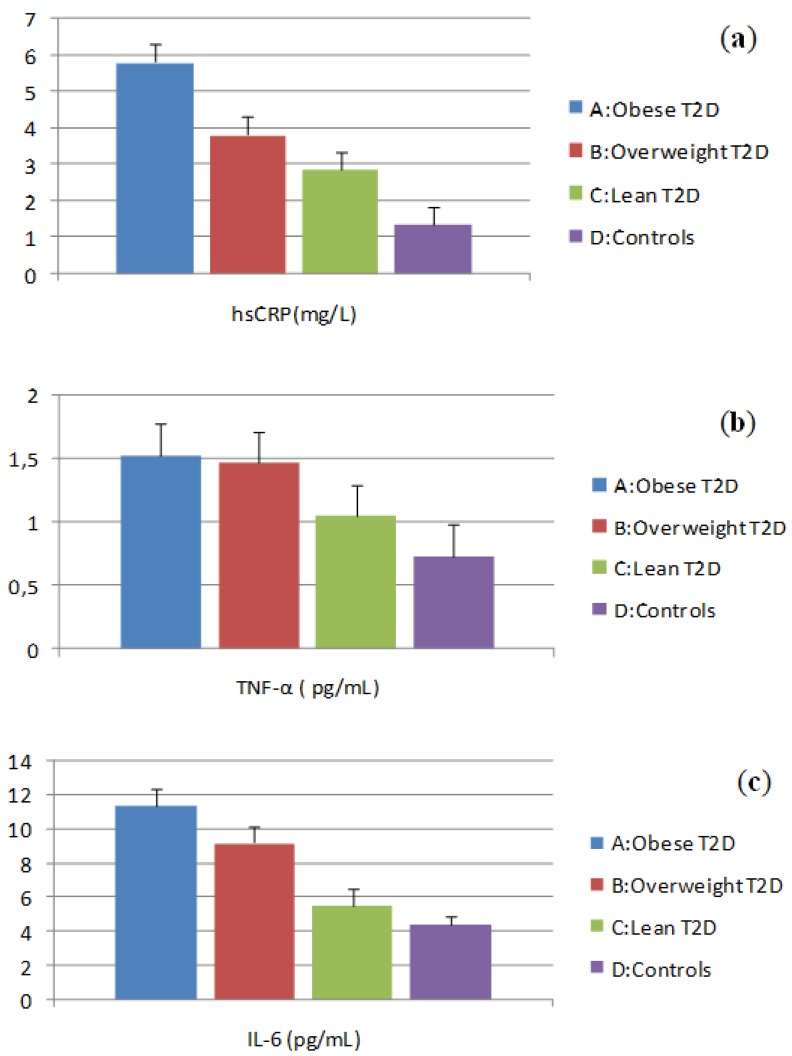
(**a**) Plasma levels of hsCRP; (**b**) Plasma levels of TNF-α; (**c**) Plasma levels of IL-6.

Notes: In (**a**): A *vs*. B: *p* < 0.01; A, B *vs*. C: *p* < 0.05; C *vs*. D: *p* < 0.01; In (**b**): A *vs*. B: *p* = NS; A, B *vs*. C: *p* < 0.01; C *vs*. D: *p* < 0.05; In (**c**): A *vs.* B: *p* < 0.05; A, B *vs*. C: *p* < 0.01; C *vs*. D: = NS.

Basal plasma levels of the inflammatory markers hsCRP, IL-6 and TNF-α are shown in Figure 3a–c. The highest levels of hsCRP were found in group A which differed significantly from group B. Both groups A and B had higher hsCRP levels compared with group C and there was a significant difference between group C and group D (Figure 3a).

The plasma TNF-α levels were the highest in group A and did not differ from group B. Group A and group B had higher TNF-α levels compared with group C, while the latter had significantly higher TNF-α levels compared to group D (Figure 3b).

Also, the highest IL-6 levels were found in group A, higher than in group B. Both groups A and B had higher IL-6 levels compared to group C. Group C IL-6 levels were not different from those in group D (Figure 3c).

### 3.4. Correlations

Correlations between adipocytokine levels, inflammatory markers and the parameters of obesity are presented in Table 2. Bivariate correlation between adipocytokines and BMI, waist circumference and fat mass that included all the subjects showed a significant negative correlation between adiponectin and these parameters of obesity. Correlations between leptin and resistin levels and obesity parameters were significant and positive. Also, inflammatory markers and the parameters of obesity exhibited a significant positive correlation.

**Table 2:** Correlation between adipocytokines and inflammatory markers and parameters of obesity.

Adipocytokines and Inflammatory Markers	BMI		Waist		Fat Mass	
	r	*p*	r	*p*	r	*p*
Adiponectin	−0.53	˂0.01	−0.60	˂0.01	−0.40	˂0.01
Leptin	0.58	˂0.01	0.35	˂0.01	0.65	˂0.01
Resistin	0.45	˂0.01	0.43	˂0.01	0.41	˂0.01
TNF-α	0.57	˂0.01	0.55	˂0.01	0.48	˂0.01
IL-6	0.57	˂0.01	0.50	˂0.01	0.51	˂0.01
hsCRP	0.29	˂0.01	0.20	˂0.05	0.32	˂0.01

For all the study participants, significance of the correlations between adipocytokine levels, inflammatory markers, plasma insulin levels and IR is presented in Table 3 and Table 4.

Among the adipocytokines, adiponectin showed a significant negative correlation with plasma insulin and HOMA-IR, while resistin exhibited a significant positive correlation with both plasma insulin and HOMA-IR. However, we could not demonstrate the significance of the correlation between leptin, plasma insulin and HOMA-IR (Table 3). Among the inflammatory markers, we found a significant positive correlation between hsCRP, TNF-α and IL-6 levels and plasma insulin and HOMA-IR (Table 4).

**Table 3:** Correlation between adipocytokines, inflammatory markers and insulin resistance.

Adipocytokinesand Inflammatory Markers	*Insulin*		*HOMA-IR*	
	r	*p*	r	*p*
Adiponectin	−0.55	<0.01	−0.57	<0.01
Leptin	0.24	NS	0.24	NS
Resistin	0.49	<0.01	0.51	<0.01
TNF-α	0.50	<0.01	0.54	<0.01
IL-6	0.33	<0.01	0.37	<0.01
hsCRP	0.31	<0.01	0.33	<0.01

When correlations between the levels of adipocytokines and inflammatory markers were analysed in T2D patients, a significant negative correlation was found only between adiponectin levels and TNF-α. Leptin levels showed a positive correlation with both IL-6 and hsCRP and resistin levels were in a significant positive correlation with TNF-α and IL-6 (Table 4).

**Table 4:** Correlation between adipocytokines and inflammatory markers in T2D patients.

Inflammatory Markers	*Adiponectin*		*Leptin*		*Resistin*	
	r	*p*	r	*p*	r	*p*
TNF-α	−0.34	<0.01	0.08	NS	0.26	<0.05
IL-6	−0.10	NS	0.35	<0.01	0.34	˂0.01
hsCRP	−0.09	NS	0.43	<0.01	0.49	NS

To adjust for potential confounders we created multivariate regression models, adjusting for successively introduced covariates: BMI, fat mass, waist circumference, HOMA-IR and insulin level. After adjustment for BMI (R^2^ = 0.88) all the positive and negative significant correlations became non- significant except a negative correlation between TNF-α and adiponectin. Our findings indicate that the correlation between TNF-α and adiponectin is independent of obesity among diabetic patients and that the correlation of leptin and resistin with the inflammatory markers is obesity-dependent. With adjustment for waist circumference (R^2^ = 0.92) we got the same results as with adjustment for BMI, indicating that abdominal obesity as well as total obesity had a strong influence on the correlation between leptin and resistin and the inflammatory markers. When we created a multivariate linear model with FM as a potential confounder (R^2^ = 0.78), we found that all the positive and negative significant correlations between adipocytokines and the inflammatory markers became non-significant, pointing to a role of adipose tissue mass in the correlation between adipocytokines and inflammatory markers. However, after adjusting for HOMA-IR and insulin (R^2^ = 0.84) all associations became non-significant, which confirmed prior findings of the strong influence of insulin resistance as a background for the interplay of adipocytokines and inflammation.

### 3.5. Discussion

In this study, we analysed the impact of obesity and T2D on adipocytokines (adiponectin, leptin and resistin) and inflammatory markers (hsCRP, TNF-α and IL-6) which leads to increased risk for CVD and might be a target for preventive obesity-reducing intervention.

The present study confirmed previous findings that obesity and T2D are associated with low plasma adiponectin concentrations. Many studies have described a significant negative correlation between BMI and plasma adiponectin levels, as well as a negative correlation between adiponectin and percent body fat, waist-to-hip ratio and intra-abdominal fat [11,12,42]. Our findings indicate that non-obese T2D patients have lower plasma adiponectin levels when compared with matched non-obese normoglycemic control subjects. Moreover, increased body weight in diabetics makes hypo-adiponectinemia more evident even among overweight T2D patients. However, hypoadiponectinemia does not become more prominent with further increases in BMI in obese T2D, which is in concordance with a number of previous studies [12,38,43,44,45,46]. As far as IR is concerned, it has been demonstrated that plasma adiponectin concentrations correlate positively with insulin sensitivity and negatively with fasting plasma insulin concentrations [10,11,47] and in some studies this correlation was independent of obesity [48]. In our study, adiponectin was found to be correlated with IR and insulinemia. Our results show that the hypoadiponectinemia in patients with obesity and T2D is more related to diabetes; however, obesity among diabetic patients causes further decreases in the adiponectin level.

Many studies in the human population have shown that obesity is generally associated with elevated leptin levels in proportion to the degree of adiposity [16]. There are a lot of conflicting results regarding the increase or decrease in leptin levels among T2D patients [49,50]. In our study, no difference in leptin levels was found between non-obese T2D patients and non-obese controls. The highest concentrations of leptin were found among obese T2D patients, and a significant difference in leptin levels was discovered between overweight and obese T2D patients. In cross-sectional studies, elevated leptin levels have been associated with IR and other comorbidities that accompany obesity such as CVD [19,20,51]. A growing body of evidence suggests that either impaired or deficient leptin signaling results in the development of IR and impaired glucose metabolism [52,53]. In our study, leptin levels did not correlate with the parameters of IR. Our data confirmed that increased leptin levels in T2D patients were more related to the degree of adiposity than to the presence of T2D.

Over the past years, some studies in humans have examined the relationship of circulating resistin levels to obesity and diabetes [34,35]. The results of these studies have been contradictory and difficult to interpret due to inconsistencies in the target epitopes used in resistin assays, as well as in ethnicity and clinical background of the subjects investigated. We demonstrated that plasma resistin concentrations are higher in non-obese T2D patients when compared with healthy non-obese control subjects. Resistin levels rise in parallel with increases in BMI in overweight and obese T2D patients, but not in proportion to the degree of adiposity. Some studies have described the same results as ours, confirming higher circulating resistin levels in diabetic patients [42,54]. Several studies showed that increased resistin in T2D was not associated with markers of insulin resistance [22,23,55]. In contrast, Silha [56] as well as Al Harithy [57] reported the same results as we did, of a significant correlation between elevated resistin levels and the HOMA-IR index in patients with T2D. Our results show that in T2D patients elevated resistin levels are more related to the diabetic state and IR than to total adiposity.

In previous studies, elevated concentrations of CRP have been associated with obesity, diabetes and CVD [27,29]. The highest levels of hsCRP in our study were found in obese T2D patients and were higher in comparison to overweight, lean diabetic patients and the control group. In our study, hsCRP levels correlated with the parameters of IR. Our data confirmed that increased hsCRP levels in obese T2D patients were related to the presence of T2D as well as to the degree of total adiposity.

We found that plasma TNF-α levels were higher in lean T2D patients in comparison to lean healthy subjects. TNF-α levels were further increased in overweight and obese diabetic patients but not in proportion to the increase in total obesity. Furthermore, we demonstrated a correlation between TNF-α levels and the parameters of IR. Nilsson reported that the plasma TNF-α concentrations were increased in lean and in obese T2D patients [58], the same results as we found. Katsuki found that the serum TNF-α concentration was elevated in obese T2D, but not in lean T2D patients [59]. Zinman reported that the serum TNF-α concentration was elevated in T2D, but after adjustment for the severity of IR (HOMA-IR) it was not higher than in control subjects [60]. The Insulin Resistance Atherosclerosis Study (IRAS) confirmed that increased TNF-α levels in T2D were predominantly associated with IR, independently of adiposity [36]. We have shown that the elevated TNF-α levels in patients with obesity and T2D are more related to the diabetic state and IR than to the degree of obesity, however, obesity among diabetic patients further increases TNF-α levels.

It has been shown that adipose tissue is a source of circulating IL-6 and a significant relation of IL-6 to BMI and adipocyte size was demonstrated [6,7]. Increased plasma levels of IL-6 were reported in obese patients as well as in T2D [31,32,33]. We found the IL-6 levels were elevated among obese diabetic patients and were higher compare with overweight T2D patients. We did not find a difference in IL-6 levels between non-obese T2D patients and non-obese healthy subjects. Some authors have reported a correlation between IL-6 and IR [34] but others failed to find such a correlation [35]. In our study, IL-6 levels were correlated with the parameters of IR. Our data have shown that increased IL-6 levels in obese T2D patients are more related to the degree of adiposity than to the presence of T2D state.

Recent studies in type 2 diabetic patients demonstrated an inverse correlation between plasma adiponectin and inflammatory markers, including hsCRP [7,16]. In our study, we confirmed the prior findings of a negative correlation between adiponectin and TNF-α. We did not find a correlation between adiponectin levels and IL-6 and hsCRP. Moreover, the correlation between TNF-α and adiponectin was independent of obesity among our diabetic patients, as has been shown in some studies [44]. Some observational studies have suggested that leptin is associated with inflammatory markers independently of fat mass [61,62,63], while others have demonstrated that this relationship is not significant or becomes non-significant with careful control for measures of body fat [15,64]. In our study, we confirmed the significant association of obesity and the degree of obesity with leptin levels. Moreover, we showed a significant correlation between leptin levels and IL-6 and hsCRP among T2D patients and demonstrated that the correlation between leptin and the inflammatory markers is dependent on obesity. These findings indicate that leptin probably mediates the effects of obesity on inflammatory markers. We found a significant correlation between resistin levels and inflammatory markers among T2D patients and demonstrated that total obesity as well as abdominal obesity had a strong influence on the correlation between resistin and inlammatory markers. Shetty and coworkers showed that resistin’s associations with inflammatory markers in diabetic patients appear to be independent of the BMI [65].

This study provides a new data about the relationship between adipocytokines and inflammatory markers and the parameters of obesity, especially with regard to the subset of non-obese T2D patients. This subset represents only 10% of all Caucasian T2D patients, and according to our data exhibits specific characteristics compared to the obese and overweight T2D patients.

## 4. Conclusions

In conclusion, the present study showed that obesity might influence CVD risk though significant changes in adipocytokines and inflammatory markers in T2D patients. Correlation between leptin and resistin and the inflammatory markers was found to be highly dependent on obesity, in contrast to the one between adiponectin and TNF-α. Thus, leptin and resistin may represent the principal mediators of the impact of obesity on the inflammatory markers involved in cardiovascular risk in T2D patients.
